# A novel splice site mutation of *CDHR1* in a consanguineous Israeli Christian Arab family segregating autosomal recessive cone-rod dystrophy

**Published:** 2012-12-01

**Authors:** Ben Cohen, Elena Chervinsky, Haneen Jabaly-Habib, Stavit A. Shalev, Daniel Briscoe, Tamar Ben-Yosef

**Affiliations:** 1 The Rappaport Family Institute for Research in the Medical Sciences, Haifa, Israel; 2 The Rappaport Faculty of Medicine, Technion-Israel Institute of Technology, Haifa, Israel; 3 Genetics Institute, Ha’Emek Medical Center, Afula, Israel; 4 Department of Ophthalmology, Ha’Emek Medical Center, Afula, Israel

## Abstract

**Purpose:**

To investigate the genetic basis for autosomal recessive cone-rod dystrophy in a consanguineous Israeli Christian Arab family.

**Methods:**

Patients underwent a detailed ophthalmic examination, including funduscopy, electroretinography (ERG), visual field testing, and optical coherence tomography. Genome-wide homozygosity mapping using a single nucleotide polymorphism array was performed to identify homozygous regions shared between the two affected individuals. Mutation screening of the underlying gene was performed with direct sequencing. In silico analysis was used to predict the effect of the mutation on splicing.

**Results:**

The family included two affected individuals. Clinical findings included progressive deterioration of visual acuity, photophobia, defective color vision, loss of central visual fields, pigmentary deposits localized mainly in the peripheral retina, a thinned and atrophic macular region, retinal vessel attenuation, absent ERG cone responses, and reduced ERG rod responses. Homozygosity mapping revealed several homozygous intervals shared among the affected individuals. One, a 12Mb interval on chromosome 10, included the *CDHR1* gene. Direct sequencing revealed a single base transversion, c.1485+2T>G, located in the conserved donor splice site of Intron 13. This mutation cosegregated with the disease in the family, and was not detected in 208 Israeli Christian Arab control chromosomes. In silico analysis predicted that this mutation eliminates the Intron 13 donor splice site.

**Conclusions:**

Only three distinct pathogenic mutations of *CDHR1* have been reported to date in patients with autosomal recessive retinal degeneration. Here we report a novel splice site mutation of *CDHR1*, c.1485+2T>G, underlying autosomal recessive cone-rod dystrophy in a consanguineous Israeli Christian Arab family. This report expands the spectrum of pathogenic mutations of the *CDHR1* gene.

## Introduction

Hereditary retinal degeneration (HRD) is a clinically and genetically heterogeneous group of diseases that cause visual loss due to progressive loss of rod and/or cone photoreceptor cells in the retina. In cone-rod dystrophy (CRD), cone involvement initially exceeds that of rods, and therefore the predominant symptoms are reduced visual acuity, photophobia, defective color vision, and decreased sensitivity in the central visual field, later followed by progressive loss in peripheral vision and night blindness. Additional ophthalmologic findings include pigment deposits visible on fundus examination, predominantly localized to the macular region. The prevalence of CRD is approximately 1/40,000 [1,2].

CRD is a heterogeneous disorder. In most patients, the disease is limited to the eye (nonsyndromic), with no extraocular manifestations. Nonsyndromic CRD can be inherited as autosomal recessive (ar), autosomal dominant (ad), or X-linked (XL). More than 20 genes and loci have been implicated in nonsyndromic CRD, of which at least six are associated with an autosomal recessive mode of inheritance (Retnet- Retinal Information Network, RetNet). One is *CDHR1.*

*CDHR1* (previously known as *PCDH21*) encodes for cadherin-related family member 1. Members of the cadherin family of transmembrane proteins are often involved in calcium-dependent cell adhesion. Cadherins are characterized by the presence of 1–34 extracellular cadherin (EC) domains (composed of about 110 amino acids), which contain highly conserved Ca^2+^-binding motifs. Variations in the cytoplasmic domains impart functional specificity by conferring to each molecule the ability to interact with different ligands (reviewed in [3]). Interestingly, four members of the cadherin family have been linked to retinal degeneration: Mutations in the genes encoding cadherin 23 (*CDH23*) and protocadherin 15 (*PCDH15*) cause Usher syndrome (a syndromic form of HRD, characterized by the combination of retinitis pigmentosa [RP] and hearing loss) [4–6], mutations of *CDH3*, encoding P-cadherin, lead to hypotrichosis with juvenile macular dystrophy [7], and mutations of *CDHR1* cause arCRD [8].

*CDHR1* consists of six EC domains with a unique intracellular domain. *CDHR1* is expressed only in a small subset of neuronal tissues, including the olfactory bulb and the retina [9,10]. In the retina, *CDHR1* is localized to the junction between the inner and outer segments of rod and cone photoreceptors, and has a crucial role in photoreceptor outer segment disc assembly. Outer segments of *CDHR1* knockout mice are disorganized, and there is progressive death of photoreceptor cells [10]. *CDHR1* was therefore an obvious candidate for human retinal degeneration. Screening of the *CDHR1* gene in a large cohort of patients with various forms of HRD led to the identification of two missense variants: p.A212T and p.P532A. Both variants affected evolutionary conserved residues, and were not detected in unaffected controls. However, since both were found in a heterozygous state and a second allele could not be identified in both cases, their pathogenicity remained uncertain [11]. Recently, three distinct pathogenic mutations of *CDHR1* have been reported in patients with CRD from the Faroe Islands, the Middle East, and South Asia [8,12] (Table 1). Here we report a novel splice site mutation of *CDHR1* underlying arCRD in a consanguineous Israeli Christian Arab family.

**Table 1:** Currently known pathogenic mutations of the CDHR1 gene

Exon/Intron	Base change	Amino acid substitution	Ethnicity	Reference
Exon 4	c.338delG	p.G113AfsX1	Middle East	[12]
Exon 6	c.524dupA	p.Q175QfsX47	Faroe Islands	[8]
Exon 13	c.1459delG	p.G487GfsX20	South Asia	[12]
Intron 13	c.1485+2T>G	Aberrant splicing	Israeli Christian Arab	Current report

## Methods

### Patients

Four members of a Christian Arab consanguineous family from northern Israel (family TB127) were ascertained for this study. The study was performed in accordance with the Declaration of Helsinki, and written informed consent was obtained from all participants. The research was approved by the local institutional review board at Ha’emek Medical Center and by the National Helsinki Committee for Genetic Research in Humans. DNA control samples were obtained from Christian Arab individuals from northern Israel who presented for routine genetic testing and consented for the use of their DNA samples in additional genetic studies.

### DNA analysis

Venous blood samples were obtained using K3EDTA vacuette tubes (Greiner Bio-One, Kremsmunster, Austria), and genomic DNA was extracted using a high salt solution according to a standard protocol [13]. Genome-wide homozygosity mapping was performed using the HumanCytoSNP-12v2.1 BeadChip (220 K; Illumina, Inc., San Diego, CA). Homozygous regions were calculated using HomozygosityMapper [14]. For mutation analysis, specific primers were used to PCR-amplify the 17 exons of *CDHR1*, including intron-exon boundaries. Primer sequences were as previously described [8,11]. Mutation screening was performed by direct sequencing with the Big Dye terminator cycle sequencing kit on an ABI 3130×l Genetic Analyzer (PE Applied Biosystems, Foster City, CA). The DNA control samples for the c.1485+2T>G mutation were screened with a restriction endonuclease-based assay with HpyCH4III (New England Biolabs, Beverly, MA). *CDHR1* exon 13 was PCR-amplified in a 25 μl reaction volume. Twenty μl of the products were digested overnight in a 30 μl volume with HpyCH4III (5 U, 37 °C) and 1X of the recommended buffer. The entire reaction volume (30 μl) was visualized with electrophoresis on a 2% agarose gel. Expected band sizes were 300 and 90 bp for the wild-type allele and 390 bp for the mutant allele.

### Splice site score predictions

The genomic sequence environment of the mutation was analyzed for 5′ and 3′ splice sites using Automated Splice Site Analyses [15] and ASD – Intron Analysis [16].

## Results

### Clinical findings

**Figure 1:**
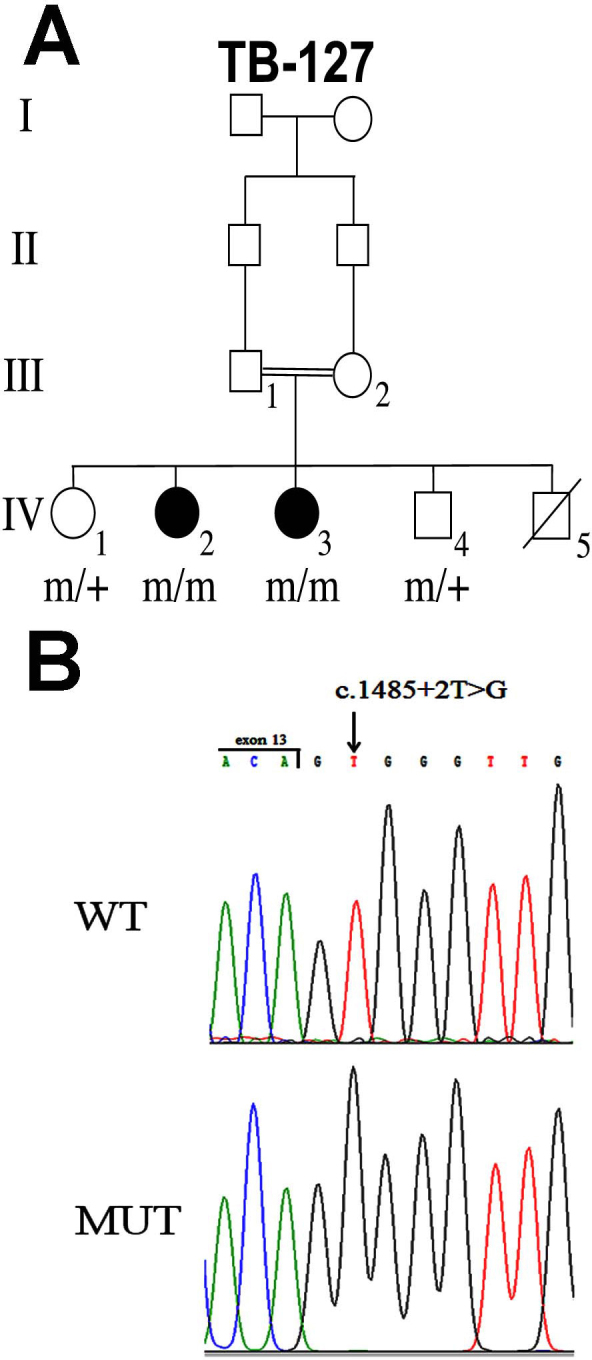
Pedigree and mutation analysis. **A**: Shown is a consanguineous Israeli Christian Arab family segregating arCRD (family TB127). Filled symbols represent affected individuals, whereas clear symbols represent unaffected individuals. Genotypes of family members at the *CDHR1* gene are indicated. m, mutant allele; +, wt allele. **B**: Nucleotide sequence traces of the boundary between the *CDHR1* exon and Intron 13 in a non-carrier individual (wt) and an affected individual homozygote for the c.1485+2T>**G**: mutant allele (mut). The exon-intron boundary is marked.

**Figure 2:**
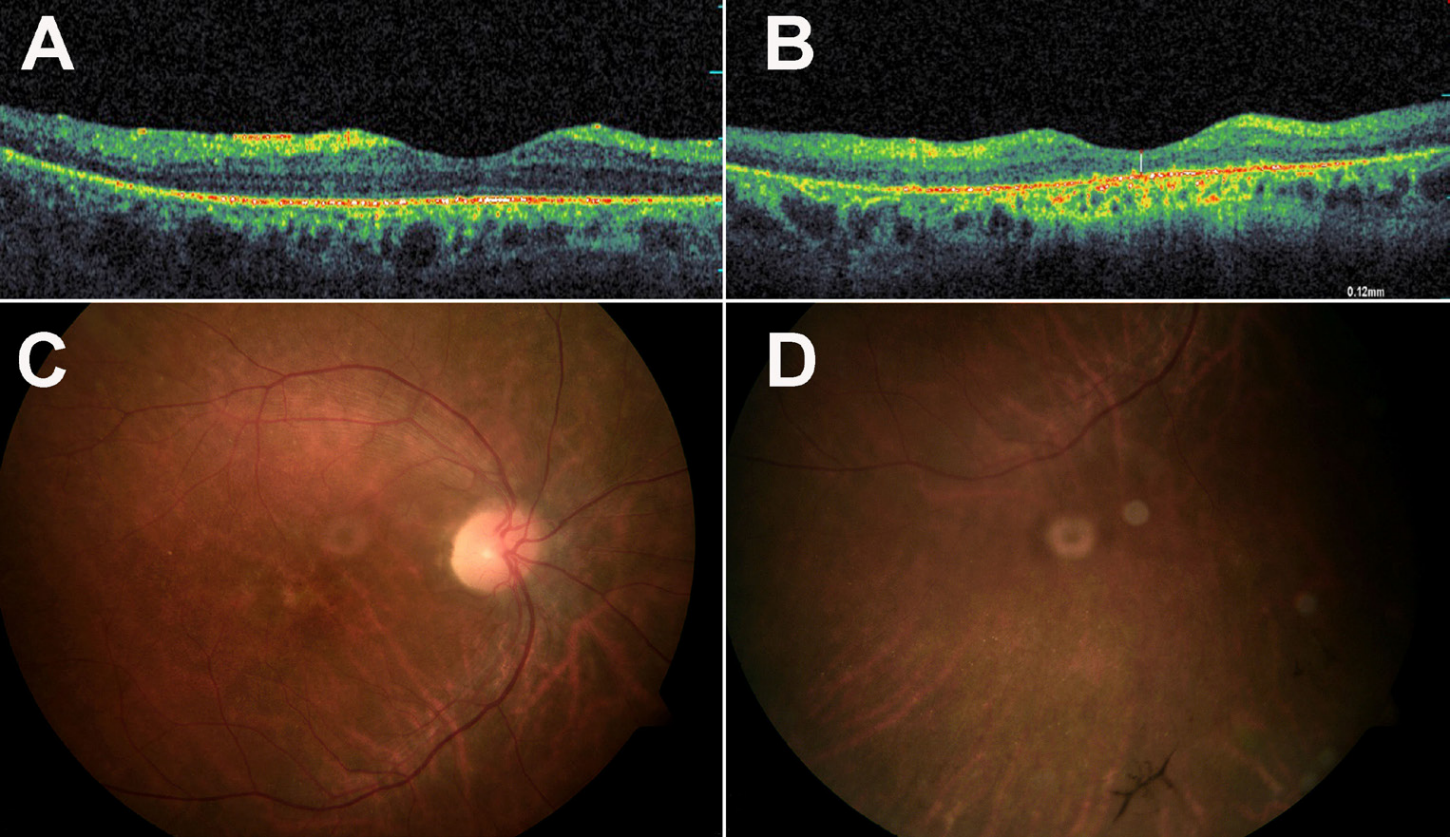
Optical coherence tomography and fundus photographs of affected individuals. **A**: Optical coherence tomography (OCT) of the right eye of individual IV:3 at the age of 38 years shows macular thinning and loss of foveal contour. **B**: OCT of the right eye of individual IV:2 at the age of 36 years shows severe thinning of the macula, with irregular retinal pigment epithelium (RPE). **C**: Fundus photograph of individual IV:3 at the age of 38 years demonstrating pink optic disc and moderately attenuated retinal vessels. Macular involvement is indicated by the lack of macular reflex, and by macular atrophy. Pigmentary changes (beaten bronze like) are observed in the macular region. **D**: Fundus photograph of retinal periphery in individual IV:3 demonstrating bone spicule-like pigmentation deposits and punctate salt- and pepper-like appearance.

Family TB127 is a Christian Arab family from northern Israel. Parents are first cousins, and two of their five offspring have CRD (Figure 1A). Impaired day vision was first noticed in their 20s. In the third decade of life, electroretinographic (ERG) cone response was absent, while rod response was markedly reduced (Table 2). Pattern visual evoked potentials were of reduced waveforms and prolonged implicit time, indicating severely reduced macular function. Flash visual evoked potentials indicated that nerve conduction of both optic nerves to the visual cortex was within normal limits (data not shown). In the fourth decade of life, visual acuity was markedly decreased, and color vision was severely impaired (Table 2). Humphrey visual field testing showed bilateral deep large central scotomas with significant general reduction of sensitivity in both eyes. Optical coherence tomography imaging showed bilateral severe thinning of the macula. For example, in patient IV:3 macular thickness in the right eye was 140 μm (normal 200 μm), and there was loss of the foveal contour. In the left eye, the macular thickness was normal, but loss of the foveal contour was more prominent than in the right eye (Figure 2A). An irregular retinal pigment epithelium layer was seen in optical coherence tomography of one patient (individual IV:2; Figure 2B). Funduscopic examination revealed pink optic discs and moderately attenuated retinal vessels. Macular involvement was indicated by the lack of macular reflex, and by macular atrophy. In patient IV:2, there were no pigmentary changes, while in patient IV:3 pigmentary changes (beaten bronze like) were observed in the macula, and in the periphery there were a few bone spicule-like pigmentation deposits and punctate salt- and pepper-like appearance (Figure 2C,D).

**Table 2:** Clinical characteristics of individuals homozygous for the CDHR1 c.1485+2T>G mutation

Patient No. Sex	Age (y)	Visual acuity	Refractive error	Color vision (Ishihara)	FFERG*				
					Age (y)	Eye	LA: single flash	LA: flicker (30Hz)	DA: (μV)║
IV-2 F	36	6/120(OU)	OD −11	1st panel not seen (OU)	23	OD	NR	ND	a 0 b 80 †
			OS −13			OS	NR	ND	a 0 b 50
IV-3 F	38	6/120(OU)	ND	1st panel not seen (OU)	30	OD	NR	NR	a 0 b 17 ‡
						OS	ND	ND	ND

F, Female; y, years; OD, right eye; OS, left eye; OU, both eyes; NR, Non-Recordable; ND, Not Determined *Full-Field Electroretinogram; LA: light adaptation (cone ERG); DA: dark adaptation (rod ERG) ║ERG recordings for the two patients were performed with different systems. Therefore, the normal range is indicated for each patient separately. †DA: Normal a-wave 260–630μV; Normal b-wave 440–800 μV ‡DA: Normal a-wave 160–320 μV; Normal b-wave 300–500 μV

### Genetic analysis

Family TB127 is consanguineous, and CRD segregates in an autosomal recessive mode. We therefore performed genome-wide homozygosity mapping using the HumanCytoSNP-12v2.1 BeadChip (220 K). Several homozygous intervals ranging from 2 to 65 Mb in size were shared between the two affected individuals (Table 3). Of the six arCRD causative genes known to date (*ABCA4* [17], *ADAM9* [18], *C8ORF37* [19], *CERKL* [20], *RPGRIP1* [21], and *CDHR1* [8]), *CDHR1* was the only one included in a homozygous interval. Several additional genes known to be involved in other forms of HRD were also located within these intervals, including the following: *GPR98,* underlying Usher syndrome type 2C [22]; *TOPORS*, underlying adRP [23]; and *RGR*, underlying arRP [24]. However, since the phenotype in both patients was consistent with a diagnosis of CRD, *CDHR1* appeared the best candidate gene in this family. Sequence analysis of the 17 coding exons of *CDHR1*, including exon-intron boundaries, was performed in one affected individual. We identified a single base transversion, c.1485+2T>G, located in the conserved donor splice site of Intron 13 (Figure 1B). This mutation was present homozygously in both affected individuals, and heterozygously in their two unaffected siblings (Figure 1A). The mutation was not detected in the 1000 Genomes Database or in 208 ethnically-matched control chromosomes, indicating that it is not a common polymorphism. This was done by restriction digest, as described in detail in the Methods section. Our Israeli patients with CRD included ten families of Christian Arab descent. In four families, pathogenic mutations of other HRD-causative genes have been detected. The c.1485+2T>G mutation of *CDHR1* was not detected in the six remaining families. These findings indicate that c.1485+2T>G is probably a rare private mutation and does not significantly contribute to the CRD phenotype among Israeli Christian Arabs.

**Table 3:** Homozygosity mapping results in family TB127

Chromosome	Interval of homozygosity (Mb)	Interval size (Mb)	Candidate genes
9	26–91	65	*TOPORS*
5	82–106	24	*GPR98*
3	133–146	13	
10	83–95	12	*CDHR1, RGR*
7	0–8	8	
10	120–124	4	
3	94–96	2	
8	19–21	2	
16	79–81	2	

### In silico analysis of the splice site mutation

According to the splice site consensus sequence in mammals, a T nucleotide is located at position +2 of the donor site [25]. Indeed, in the wild-type *CDHR1* allele a T is located at position +2 of intron 13. However, in the mutant allele the nucleotide at position +2 is G (Figure 1B). To predict the effect of this transversion on splicing, we performed in silico analysis of the sequence using two different web-based tools (Automated Splice Site Analyses and ASD – Intron Analysis). Both algorithms predicted that the c.1485+2T>G mutation leads to elimination of the Intron 13 donor site (Table 4 and data not shown).

**Table 4:** Analysis of CDHR1 intron 13 donor splice-site and adjacent potential sites by the Automated Splice Site Analyses tool

Genomic coordinate*	wt sequence	Mutant sequence	Position relative to natural site	Initial score (Ri)†	Final score (Ri)‡	ΔRi║
85,970,922–85970923	acagtggg	acaggggg	0	4.0	−4.2	−8.2
85,970,950–85970951	tgggtggg	NA	+27	3.8	3.8	0.0

NA, not applicable *Position of donor site according to human genome reference sequence (hg19) †Initial score measured at the base before the mutation is made ‡ Final score measured at the base after the mutation is made ║Final (Ri)-Initial (Ri)

*CDHR1* cDNA (GenBank accession number NM_033100.2) encodes for a protein of 859 amino acids, which includes six EC domains, a transmembrane domain, and a unique intracellular domain. The exact effect of the c.1485+2T>G splicing mutation on *CDHR1* transcripts in vivo is not known. One option is retention of the entire Intron 13, which is expected to yield a protein of 548 amino acids, in which the last 54 amino acids are incorrect. Another option is that elimination of the natural Intron 13 donor splice site leads to the use of a cryptic donor site. Analysis of the *CDHR1* gene with the Automated Splice Site Analyses tool indicated the presence of a relatively strong cryptic donor site located 27 bp downstream of the native splice site within Intron 13 (Table 4). The use of this cryptic site is expected to yield a protein of 543 amino acids, in which the last 49 amino acids are incorrect. Both options yield proteins in which two EC domains, the transmembrane domain, and the entire intracellular domain are missing.

## Discussion

The aim of the current study was to investigate the genetic basis for arCRD in a consanguineous Israeli Christian Arab family. Genetic analysis revealed a novel splice site mutation of *CDHR1*, c.1485+2T>G. We performed in silico analysis, which demonstrated that the Intron 13 donor splice site harboring this mutation is not efficiently recognized by the human splicing machinery. Although the exact effect of this splicing mutation on *CDHR1* transcripts in vivo is not known, the expected outcome is incorrect splicing, leading to an abnormal protein product.

The phenotype associated with *CDHR1* mutations reported to date is consistent with a diagnosis of arCRD. In general, clinical findings in affected individuals from family TB127 are similar to those previously reported in patients with other *CDHR1* mutations. These include progressive deterioration of visual acuity, photophobia, defective color vision, loss of central visual fields, macular atrophy, retinal vessel attenuation, and reduced or absent ERG responses. Interestingly, in previously reported cases pigment deposits were predominantly localized to the macular region. This was not seen in our patients. Pigmentary deposits were detected in one patient only and were localized mainly to the peripheral region [8,12] (Table 2 and Figure 2).

To date, six arCRD causative genes have been identified. In a recent survey of a large CRD cohort from the Netherlands, *ABCA4* mutations were found in 26% of the patients with arCRD [2]. In contrast, mutations in other arCRD genes are rare, and each one is found in only a few families worldwide. For example, only eight mutations have been reported for *CERKL* [20,26–30], four mutations for *ADAM9* [18], and three mutations for *C8ORF37* [19]. Since a pathogenic *CDHR1* mutation was identified in a family from the Faroe Islands in 2010, only two additional mutations have been reported [8,12] (Table 1). This small number suggests that mutations in this gene are a rare cause of arCRD. Here we report a consanguineous Israeli Christian Arab family segregating arCRD due to a novel splice site mutation of *CDHR1*. This report expands the spectrum of pathogenic mutations of the *CDHR1* gene.

## References

[1] Hamel CP (2007). Cone rod dystrophies.. Orphanet J Rare Dis.

[2] Thiadens AA, Phan TM, Zekveld-Vroon RC, Leroy BP, van den Born LI, Hoyng CB, Klaver CC, Roosing S, Pott JW, van Schooneveld MJ, van Moll-Ramirez N, van Genderen MM, Boon CJ, den Hollander AI, Bergen AA, De Baere E, Cremers FP, Lotery AJ (2012). Clinical Course, Genetic Etiology, and Visual Outcome in Cone and Cone-Rod Dystrophy.. Ophthalmology.

[3] Shapiro L, Weis WI (2009). Structure and biochemistry of cadherins and catenins.. Cold Spring Harb Perspect Biol.

[4] Ahmed ZM, Riazuddin S, Bernstein SL, Ahmed Z, Khan S, Griffith AJ, Morell RJ, Friedman TB, Wilcox ER (2001). Mutations of the protocadherin gene PCDH15 cause Usher syndrome type 1F.. Am J Hum Genet.

[5] Bolz H, von Brederlow B, Ramirez A, Bryda EC, Kutsche K, Nothwang HG, Seeliger M (2001). del CSCM, Vila MC, Molina OP, Gal A, Kubisch C. Mutation of CDH23, encoding a new member of the cadherin gene family, causes Usher syndrome type 1D.. Nat Genet.

[6] Bork JM, Peters LM, Riazuddin S, Bernstein SL, Ahmed ZM, Ness SL, Polomeno R, Ramesh A, Schloss M, Srisailpathy CR, Wayne S, Bellman S, Desmukh D, Ahmed Z, Khan SN, Kaloustian VM, Li XC, Lalwani A, Bitner-Glindzicz M, Nance WE, Liu XZ, Wistow G, Smith RJ, Griffith AJ, Wilcox ER, Friedman TB, Morell RJ (2001). Usher syndrome 1D and nonsyndromic autosomal recessive deafness DFNB12 are caused by allelic mutations of the novel cadherin-like gene CDH23.. Am J Hum Genet.

[7] Sprecher E, Bergman R, Richard G, Lurie R, Shalev S, Petronius D, Shalata A, Anbinder Y, Leibu R, Perlman I, Cohen N, Szargel R (2001). Hypotrichosis with juvenile macular dystrophy is caused by a mutation in CDH3, encoding P-cadherin.. Nat Genet.

[8] Ostergaard E, Batbayli M, Duno M, Vilhelmsen K, Rosenberg T (2010). Mutations in PCDH21 cause autosomal recessive cone-rod dystrophy.. J Med Genet.

[9] Nakajima D, Nakayama M, Kikuno R, Hirosawa M, Nagase T, Ohara O (2001). Identification of three novel non-classical cadherin genes through comprehensive analysis of large cDNAs.. Brain Res Mol Brain Res.

[10] Rattner A, Smallwood PM, Williams J, Cooke C, Savchenko A, Lyubarsky A, Pugh EN, Nathans J (2001). A photoreceptor-specific cadherin is essential for the structural integrity of the outer segment and for photoreceptor survival.. Neuron.

[11] Bolz H, Ebermann I, Gal A (2005). Protocadherin-21 (PCDH21), a candidate gene for human retinal dystrophies.. Mol Vis.

[12] Henderson RH, Li Z, Abd El Aziz MM, Mackay DS, Eljinini MA, Zeidan M, Moore AT, Bhattacharya SS, Webster AR (2010). Biallelic mutation of protocadherin-21 (PCDH21) causes retinal degeneration in humans.. Mol Vis.

[13] Grimberg J, Nawoschik S, Belluscio L, McKee R, Turck A, Eisenberg A (1989). A simple and efficient non-organic procedure for the isolation of genomic DNA from blood.. Nucleic Acids Res.

[14] Seelow D, Schuelke M, Hildebrandt F, Nürnberg P (2009). HomozygosityMapper–an interactive approach to homozygosity mapping.. Nucleic Acids Res.

[15] Nalla VK, Rogan PK (2005). Automated splicing mutation analysis by information theory.. Hum Mutat.

[16] Stamm S, Riethoven JJ, Le Texier V, Gopalakrishnan C, Kumanduri V, Tang Y, Barbosa-Morais NL, Thanaraj TA (2006). ASD: a bioinformatics resource on alternative splicing.. Nucleic Acids Res.

[17] Cremers FP, van de Pol DJ, van Driel M, den Hollander AI, van Haren FJ, Knoers NV, Tijmes N, Bergen AA, Rohrschneider K, Blankenagel A, Pinckers AJ, Deutman AF, Hoyng CB (1998). Autosomal recessive retinitis pigmentosa and cone-rod dystrophy caused by splice site mutations in the Stargardt's disease gene ABCR.. Hum Mol Genet.

[18] Parry DA, Toomes C, Bida L, Danciger M, Towns KV, McKibbin M, Jacobson SG, Logan CV, Ali M, Bond J, Chance R, Swendeman S, Daniele LL, Springell K, Adams M, Johnson CA, Booth AP, Jafri H, Rashid Y, Banin E, Strom TM, Farber DB, Sharon D, Blobel CP, Pugh EN, Pierce EA, Inglehearn CF (2009). Loss of the metalloprotease ADAM9 leads to cone-rod dystrophy in humans and retinal degeneration in mice.. Am J Hum Genet.

[19] Estrada-Cuzcano A, Neveling K, Kohl S, Banin E, Rotenstreich Y, Sharon D, Falik-Zaccai TC, Hipp S, Roepman R, Wissinger B, Letteboer SJ, Mans DA, Blokland EA, Kwint MP, Gijsen SJ, van Huet RA, Collin RW, Scheffer H, Veltman JA, Zrenner E, den Hollander AI, Klevering BJ, Cremers FP (2012). Mutations in C8orf37, encoding a ciliary protein, are associated with autosomal-recessive retinal dystrophies with early macular involvement.. Am J Hum Genet.

[20] Aleman TS, Soumittra N, Cideciyan AV, Sumaroka AM, Ramprasad VL, Herrera W, Windsor EA, Schwartz SB, Russell RC, Roman AJ, Inglehearn CF, Kumaramanickavel G, Stone EM, Fishman GA, Jacobson SG (2009). CERKL mutations cause an autosomal recessive cone-rod dystrophy with inner retinopathy.. Invest Ophthalmol Vis Sci.

[21] Hameed A, Abid A, Aziz A, Ismail M, Mehdi SQ, Khaliq S (2003). Evidence of RPGRIP1 gene mutations associated with recessive cone-rod dystrophy.. J Med Genet.

[22] Weston MD, Luijendijk MW, Humphrey KD, Moller C, Kimberling WJ (2004). Mutations in the VLGR1 gene implicate G-protein signaling in the pathogenesis of Usher syndrome type II.. Am J Hum Genet.

[23] Chakarova CF, Papaioannou MG, Khanna H, Lopez I, Waseem N, Shah A, Theis T, Friedman J, Maubaret C, Bujakowska K, Veraitch B, Abd El-Aziz MM (2007). Prescott de Q, Parapuram SK, Bickmore WA, Munro PM, Gal A, Hamel CP, Marigo V, Ponting CP, Wissinger B, Zrenner E, Matter K, Swaroop A, Koenekoop RK, Bhattacharya SS. Mutations in TOPORS cause autosomal dominant retinitis pigmentosa with perivascular retinal pigment epithelium atrophy.. Am J Hum Genet.

[24] Morimura H, Saindelle-Ribeaudeau F, Berson EL, Dryja TP (1999). Mutations in RGR, encoding a light-sensitive opsin homologue, in patients with retinitis pigmentosa.. Nat Genet.

[25] Mount SM (1982). A catalogue of splice junction sequences.. Nucleic Acids Res.

[26] Ali M, Ramprasad VL, Soumittra N, Mohamed MD, Jafri H, Rashid Y, Danciger M, McKibbin M, Kumaramanickavel G, Inglehearn CF (2008). A missense mutation in the nuclear localization signal sequence of CERKL (p.R106S) causes autosomal recessive retinal degeneration.. Mol Vis.

[27] Auslender N, Sharon D, Abbasi AH, Garzozi HJ, Banin E, Ben-Yosef T (2007). A common founder mutation of CERKL underlies autosomal recessive retinal degeneration with early macular involvement among Yemenite Jews.. Invest Ophthalmol Vis Sci.

[28] Littink KW, Koenekoop RK, van den Born LI, Collin RW, Moruz L, Veltman JA, Roosing S, Zonneveld MN, Omar A, Darvish M, Lopez I, Kroes HY, van Genderen MM, Hoyng CB, Rohrschneider K, van Schooneveld MJ, Cremers FP, den Hollander AI (2010). Homozygosity mapping in patients with cone-rod dystrophy: novel mutations and clinical characterizations.. Invest Ophthalmol Vis Sci.

[29] Tang Z, Wang Z, Ke T, Wang QK, Liu M (2009). Novel compound heterozygous mutations in CERKL cause autosomal recessive retinitis pigmentosa in a nonconsanguineous Chinese family.. Arch Ophthalmol.

[30] Tuson M, Marfany G, Gonzalez-Duarte R (2004). Mutation of CERKL, a novel human ceramide kinase gene, causes autosomal recessive retinitis pigmentosa (RP26).. Am J Hum Genet.

